# Trends and variation in urgent referrals for suspected cancer 2009/2010–2019/2020

**DOI:** 10.3399/bjgp22X718217

**Published:** 2021-12-31

**Authors:** Lesley Smith, Nigel Sansom, Scott Hemphill, Stephen H Bradley, Bethany Shinkins, Pete Wheatstone, Willie Hamilton, Richard D Neal

**Affiliations:** Division of Primary Care, Public Health & Palliative Care, Leeds Institute of Health Sciences, University of Leeds, Leeds; Leeds Centre for Personalised Medicine and Health, University of Leeds, Leeds.; PinPoint Data Science Ltd.; Division of Primary Care, Public Health & Palliative Care, Leeds Institute of Health Sciences, University of Leeds, Leeds.; Division of Primary Care, Public Health & Palliative Care, Leeds Institute of Health Sciences, University of Leeds, Leeds.; Test Evaluation Group, Leeds Institute of Health Sciences, University of Leeds.; Leeds.; University of Exeter, Exeter.; Division of Primary Care, Public Health & Palliative Care, Leeds Institute of Health Sciences, University of Leeds, Leeds; University of Exeter, Exeter.

## BACKGROUND

In the UK, the majority of cancers are diagnosed following referral from the GP after presenting with symptoms, signs, or test results associated with undiagnosed cancer.^1^ In England and Wales, an urgent referral pathway for patients presenting to GPs with ‘red flag’ symptoms was introduced in 2000, whereby all patients should be seen by a specialist within 2 weeks of referral (known as the Two Week Wait [TWW] pathway). These guidelines were updated in 2015 by incorporating more non-specific symptoms and lowering the threshold of risk of cancer from 5% to 3%.^2^ The TWW pathway is a high-volume referral pathway and the number of referrals has increased substantially over the last decade; in 2019/2020, 2.4 million patients were referred via this pathway.^3^ In addition to monitoring TWW referral rates, other key outcome indicators are the conversion rate (percentage of TWW referrals resulting in a cancer diagnosis) and the detection rate (percentage of new cancer cases treated resulting from a TWW referral) (Box 1). Improving the detection rate is a key priority as this means that patients may be diagnosed more promptly rather than by other routes such as emergency presentations, which have poorer survival.^1^ However, obtaining the right balance between high detection rate and low conversion rate is difficult and complex, involving many stakeholders.^4^ The full impact of increasing referrals on conversion and detection rates, as well as the wider impact on patients and the health system, is not well understood.^4^ There is a growing body of published data on cancer diagnostics publicly available including data by cancer pathway.

**Box 1.** Definitions of referral metrics

Two Week Wait (TWW) referral rate: number of TWW referrals divided by the population multiplied by 100 000. Conversion rate: percentage of TWW referrals that resulted in a diagnosis of cancer (equivalent to the positive predictive value for cancer among patients selected for urgent referral). Detection rate: percentage of new cancer cases treated resulting from a TWW referral (equivalent to the sensitivity of selection of patients for urgent referral).

Differences by cancer type will be influenced by the nature and presentation of cancer symptoms, changes in lifestyle risk factors (such as smoking, alcohol, and obesity) contributing to changing incidence rates, and other pathways and routes to diagnosis. In this article we review national data on the process metrics for cancer referrals by pathway, including trends over the last decade, discussing these in the context of changes to health policy and practice in England.

## TRENDS IN REFERRAL RATE METRICS

We examined publicly available data on TWW referral, conversion, and detection rates for England available from 2009/2010 to 2019/2020^3^ published by the National Cancer Registration and Analysis Service. We analysed annual trends in three referral metrics (TWW, conversion, and detection) across 11 pathways for England^3^ to estimate the annual average percentage change in each metric from 2009/2010 to 2019/2020.

**Figure 1.**
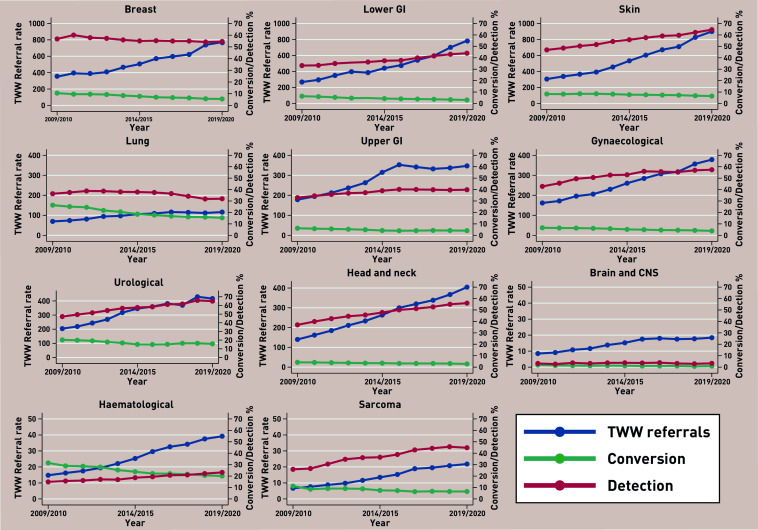
*Two Week Wait (TWW) referral, conversion, and detection rates by cancer pathway, England 2009/2010 to 2019/2020.* ***Source: National Cancer Registration and Analysis Service.****^3^* ***Contains public sector information licensed under the Open Government Licence v3.0. CNS = central nervous system. GI = gastrointestinal.***

The total number of referrals for all cancers combined increased from 902 943 in 2009/2010 to 2 374 718 in 2019/2020, corresponding to an average annual increase of 9.4% per year (95% confidence interval [CI] = 8.5 to 10.4). Increases in the referral rates were observed for all cancer pathways, ranging from 5.4% for lung (95% CI = 4.0 to 6.8) to 12.9% for sarcoma (95% CI = 12.2 to 13.7) (Table 1, Figure 1).

**Table 1.** Number and rate per 100 000 for Two Week Wait (TWW) referrals and percentages for conversion and detection rates in 2009/2010 and 2019/2020, annual average percentage change (AAPC) 2009/2010–2019/2020 by cancer pathway

**Cancer**	**TWW referrals**				**Conversion**				**Detection**		
		
	**2009/2010**		**2019/2020**		**2009/2010**		**2019/2020**		**2009/2010**	**2019/2020**	
		
	***N***	**Rate (per 100 000)**	***N***	**Rate (per 100 000)**	**AAPC (95% CI)**	***%***	***%***	**AAPC (95% CI)**	***%***	***%***	**AAPC (95% CI)**
All cancers	902 943	1729.9	2 374 718	4218.9	9.4 (8.5 to 10.4)	10.8	6.6	−4.7 (−5.2 to −4.3)	42.3	53.5	2.4 (2.1 to 2.7)
Breast	353 441	677.2	609 047	1082.0	4.5 (3.6 to 5.5)	6.3	4.3	−3.8 (−4.5 to −3.2)	56.8	54.6	−0.8 (−1.2 to −0.4)
Lower GI	140 260	268.7	441 689	784.7	10.9 (9.9 to 12.0)	6.4	3.1	−6.6 (−7.3 to −6.0)	33.2	44.0	3.0 (2.6 to 3.4)
Skin	159 430	305.4	506 456	899.8	11.8 (11.1 to 12.6)	8.3	6.5	−2.2 (−3.3 to −1.2)	46.9	64.6	3.1 (2.9 to 3.3)
Lung	36 296	69.5	65 362	116.1	5.4 (4.0 to 6.8)	26.4	15.3	−5.5 (−6.6 to −4.3)	36.4	32.0	−1.6 (−2.6 to −0.7)
Upper GI	93 048	178.3	195 353	347.1	7.0 (5.7 to 8.4)	6.2	4.1	−4.1 (−5.6 to −2.6)	33.0	39.9	1.8 (1.4 to 2.2)
Gynaecological	84 379	161.7	213 049	378.5	9.1 (8.0 to 10.2)	6.6	3.9	−5.4 (−6.0 to −4.7)	42.8	57.4	2.8 (2.0 to 3.7)
Urological	106 443	203.9	234 590	416.8	7.9 (5.9 to 9.9)	20.3	15.7	−2.7 (−4.0 to −1.3)	47.2	65.0	3.2 (2.7 to 3.6)
Head and neck	73 079	140.0	227 665	404.5	10.8 (10.1 to 11.4)	4.2	2.8	−3.6 (−4.1 to −3.1)	37.0	56.0	4.2 (3.6 to 4.9)
Brain and CNS	4461	8.5	10 355	18.4	8.2 (6.9 to 9.5)	1.9	0.9	−6.6 (−9.0 to −4.3)	3.2	3.3	0.2 (−2.4 to 3.0)
Haematological	7750	14.8	22 076	39.2	10.8 (9.4 to 12.3)	31.5	20.1	−4.4 (−5.3 to −3.5)	14.9	23.2	4.6 (4.2 to 5.0)
Sarcoma	3507	6.7	12 268	21.8	12.9 (12.2 to 13.7)	11.3	6.5	−4.9 (−6.4 to −3.4)	25.9	44.8	5.4 (3.4 to 7.5)

*Source: National Cancer Registration and Analysis Service.^3^ Contains public sector information licensed under the Open Government Licence v3.0. Annual trends from 2009/2010 to 2019/2020 were analysed to estimate the AAPC in rates. CI = confidence interval. CNS = central nervous system. GI = gastrointestinal.*

Previous research has shown that GP practices with higher TWW referral rates have been associated with lower mortality for several types of cancer and a reduction in the number of late-stage cancers detected,^5^^,^^6^ and more recently that higher cancer detection rates were associated with larger practices and those with younger GPs.^7^

## CONVERSION AND DETECTION RATE TRENDS

For all cancers combined, the conversion rate decreased from 10.8% in 2009/2010 to 6.6% in 2019/2020. Decreases in conversion rates were observed for all cancer types ranging from a 2.2% per year decrease for skin cancer (95% CI = −3.3 to −1.2) to 7.0% per year decrease for lower gastrointestinal (GI) and brain cancers: lower GI −6.6 (95% CI = −7.3 to −6.0) and brain −6.6 (95% CI = −9.0 to −4.3)) (Table 1, Figure 1).

Overall the detection rate increased from 42.3% to 53.5%. Trends in the detection rate by pathway were less consistent, ranging from a 5.4% per year increase for sarcomas (95% CI = 3.4 to 7.5) to a 1.6% per year decrease for lung cancer (95% CI = −2.6 to −0.7).

**Figure 2.**
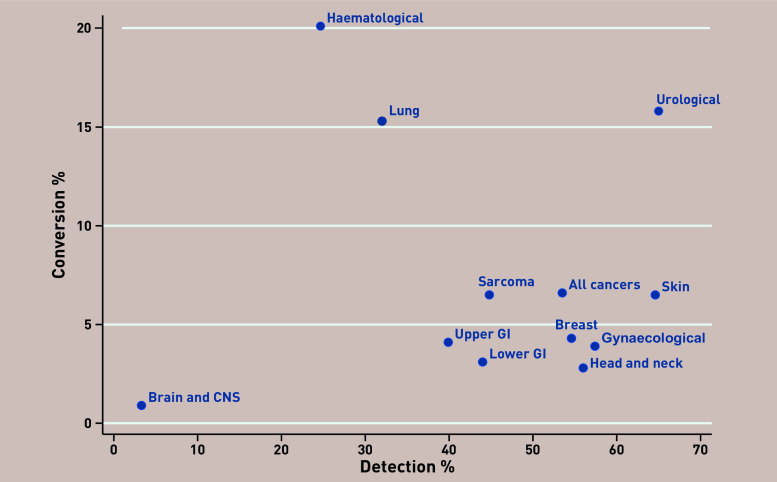
***Two Week Wait referral, conversion, and detection rates by cancer pathway, England 2009/2010 to 2019/2020. Source: National Cancer Registration and Analysis Service.****^3^* ***Contains public sector information licensed under the Open Government Licence v3.0. CNS = central nervous system. GI = gastrointestinal.***

The range in detection rate varied substantially by cancer pathway, from 3% for brain and central nervous system (CNS) tumours to 65% for urological and skin cancers (Figure 2). This variation reflects differences in the nature of cancer symptoms, presentation within primary care, and the difficulties in diagnosing some cancers. For example, both the conversion and detection rate for brain and CNS cancers were low with little change over time; this is a difficult cancer to diagnose in primary care given few low-risk features and no primary care tests.

## PATHWAYS WITH HIGH CONVERSION RATES

Cancer pathways with the highest conversion rates were lung (15%), urological (16%), and haematological (20%) cancers, which all have good triage tools within primary care.

Despite the high conversion rate, the detection rate for lung cancer was lower than for many other pathways and showed a small decrease over time; many lung cancer patients are still diagnosed via emergency routes at late stage with poorer outcomes.^1^ Urgent referrals for suspected lung cancer are frequently made after an abnormal chest X-ray suggestive of lung cancer. It is possible that local initiatives which facilitate direct referral to suspected cancer clinics upon review of abnormal chest X-ray, without requiring a formal GP TWW referral, could help explain the lower rates of change in referral rates. Chest X-rays do not initially detect a fifth of lung cancers^8^ and patients presenting with unexplained haemoptysis should be urgently referred regardless of X-ray results.^9^

Urological cancers had both high conversion and detection rates. The majority of referrals for urological cancer are for prostate cancer, which has a higher conversion rate than other urological cancers. Urgent referrals for prostate cancer are recommended in primary care for men with raised PSA levels, although the limitations of PSA testing, including high false-positive and false-negative rates, are well recognised.^10^ Several cancer awareness campaigns for bladder and kidney cancer focusing on haematuria ran from 2013–2016, which resulted in a significant increase in primary care attendance and urgent referrals.^11^ Haematological malignancies also had a high conversion rate (but low detection rate). In many cases leukaemia is readily detected by abnormalities on full blood count. However, the low detection rate indicates that for certain haematological cancers, for example, myeloma, diagnosis in primary care is difficult because of non-specific symptoms, each with low predictive power.

## PATHWAYS WITH HIGH DETECTION RATES

Urological and skin cancers had the highest detection rates. Skin cancer referrals increased by 12% per year. In 2019/2020, they accounted for 20% of all referrals with a detection rate of 65%. The increasing referral and detection rates reflect multiple factors involving patients and clinicians: greater public awareness, lower thresholds to seek medical advice, lower thresholds for referral, biopsy, and diagnosis of malignant disease.^12^ GP use of teledermatology is becoming increasingly widespread in managing TWW referrals, whereby images of the skin lesions are sent to specialists for advice prior to referral. A 2018 Cochrane Review to assess the diagnostic accuracy of teledermatology found that this method correctly identified the majority of malignant lesions and is therefore a helpful triage tool for GPs. However, further research is needed to establish the best way of providing teledermatology services.^13^ This is vitally important given the large increase in the number of referrals.

## BALANCE OF CONVERSION AND DETECTION RATES

The intention of the TWW pathway is that the majority of cancers should be diagnosed via this route. High conversion rates imply efficient use of the TWW pathway and referral of the ‘right’ patients. Obtaining the right balance between high detection rate and low conversion rate is difficult and involves many competing factors that vary by stakeholder. The observed variations in conversion and detection by cancer pathway are influenced by many factors including different symptom signatures of each cancer,^14^ cancer awareness campaigns,^11^ and available triage tools in primary care prior to referral. Economic evaluation, a quantitative framework for synthesising evidence on the implications of an intervention on patient outcomes and healthcare resource use, could provide a mechanism for identifying the optimal trade-off between referral, conversion, and detection rates. The notable differences between cancers observed highlight the need to evaluate cost-effectiveness and capacity implications of the TWW pathway for each cancer individually.

A barrier to conducting this type of analysis robustly has been the lack of evidence on the downstream impact of delays to treatment in a symptomatic population on patient outcomes and health resource use, although evidence is beginning to emerge.^5^ Monitoring the impact of delays due to the COVID-19 pandemic may provide further insight; however, data on definitive endpoints such as survival will take years to accrue.

## CHANGES TO NICE POLICY

In 2015, the National Institute for Health and Care Excellence (NICE) published NG12, which used primary care evidence to underpin its recommendations.^2^ Prior to this, NICE guidance for gynaecological cancers was updated in 2011 with the introduction of CA125 to triage women with suspected ovarian cancer. This test has been shown to be a useful triage tool in primary care settings, particularly in women aged ≥50 years and in identifying the possibility of non-ovarian cancers in women with high CA125 levels.^15^ Normal CA125 levels do not rule out cancer, with a study reporting that women with normal CA125 levels took longer to receive a diagnosis after testing but were more likely to have early-stage disease.^16^

The NICE guidance was updated in 2017, recommending the use of faecal immunochemical tests (FIT) in primary care for triaging people without rectal bleeding who have unexplained symptoms but who do not meet the criteria for an urgent referral. Evidence is emerging that this is performing well as an appropriate triage tool in primary care.^17^^,^^18^

## IMPACT OF THE COVID-19 PANDEMIC ON CANCER DIAGNOSIS

Diagnostic services have seen a substantial rise in referrals, and NHS resources may need to adapt further to increasing TWW referral rates, particularly in response to the COVID-19 pandemic and dealing with the backlog of referrals. There was a dramatic reduction in urgent suspected cancer referrals during the COVID-19 pandemic, with the number of referrals now back to pre-pandemic levels but this varies by pathway.^19^ Long-term monitoring of these trends is essential to assess the impact of the pandemic on cancer outcomes fully. It is estimated the delays to treatment of 2–6 months will lead to a substantial number of patients with early-stage tumours progressing from having curable to incurable disease.^20^ The COVID-19 pandemic has also highlighted the need to improve tests available to GPs and implement alternative strategies to better identify the patients to prioritise for referrals.

## RECOMMENDATIONS FOR FUTURE RESEARCH

These trends are based on high-quality national administrative data over a 10-year period. However, further breakdown of these data is needed to fully understand and explain these trends. The grouping together of several heterogeneous cancers within the same pathway makes it difficult to disentangle and interpret these trends. For example, head and neck cancers contain a heterogeneous mix of cancers with different referral routes. Further analysis by cancer type is needed in addition to breakdown by patient case-mix (age, sex, ethnicity, and socioeconomic status), which will influence symptom presentation, health-seeking behaviours, and GP referral patterns. It is important to continue to monitor site-specific pathways to understand the reasons for referrals, compliance with existing guidelines, and impacts of other pathways, including Rapid Diagnostic Centres and referrals for symptomatic patients who do not qualify for an urgent TWW referral. Linkage to patient outcomes, such as stage and survival, is essential to understand fully the impact of these changing trends and effectiveness of the TWW pathway.

## CONCLUSION

Referral rates have increased across all pathways; however, substantial differences in the resulting conversion and detections rates remain, reflecting differences in the nature of cancer symptoms, presentation within primary care, and the difficulties in diagnosing some cancers. Conversion rates were generally higher for cancers with good triage tools available in primary care. The impact of COVID-19 on cancer diagnosis has been substantial and highlighted the need to increase the availability of tests to GPs and implement alternative strategies to identify patients to prioritise for urgent referral.
